# Criterion-free measurement of motion transparency perception at different speeds

**DOI:** 10.1167/18.4.5

**Published:** 2018-04-03

**Authors:** Francesca Rocchi, Timothy Ledgeway, Ben S. Webb

**Affiliations:** francesca.rocchi@ncl.ac.uk; Visual Neuroscience Group, School of Psychology, University of Nottingham, Nottingham, UK; Visual Neuroscience Group, School of Psychology, University of Nottingham, Nottingham, UK; Visual Neuroscience Group, School of Psychology, University of Nottingham, Nottingham, UK

**Keywords:** *motion transparency*, *odd-one-out*, *segmentation*, *global motion*, *speed perception*

## Abstract

Transparency perception often occurs when objects within the visual scene partially occlude each other or move at the same time, at different velocities across the same spatial region. Although transparent motion perception has been extensively studied, we still do not understand how the distribution of velocities within a visual scene contribute to transparent perception. Here we use a novel psychophysical procedure to characterize the distribution of velocities in a scene that give rise to transparent motion perception. To prevent participants from adopting a subjective decision criterion when discriminating transparent motion, we used an “odd-one-out,” three-alternative forced-choice procedure. Two intervals contained the standard—a random-dot-kinematogram with dot speeds or directions sampled from a uniform distribution. The other interval contained the comparison—speeds or directions sampled from a distribution with the same range as the standard, but with a notch of different widths removed. Our results suggest that transparent motion perception is driven primarily by relatively slow speeds, and does not emerge when only very fast speeds are present within a visual scene. Transparent perception of moving surfaces is modulated by stimulus-based characteristics, such as the separation between the means of the overlapping distributions or the range of speeds presented within an image. Our work illustrates the utility of using objective, forced-choice methods to reveal the mechanisms underlying motion transparency perception.

## Introduction

A fundamental problem faced by the visual system is to parse the world into distinct objects, surfaces, and boundaries based on cues such as luminance discontinuities, wavelength, motion, and texture. The relative movement between different regions within a scene, for example, is an important source of information about relative depth and spatial structure (Wallach & O'Connell, 1953; Rogers & Graham, 1979). However, a potentially more challenging situation for vision arises when multiple objects move, at the same time, across the same region of space. This situation occurs in the natural world when moving objects partially occlude each other (e.g., a tiger moving through undergrowth) or are viewed through a semitransparent medium (e.g., a fish swimming against the current in a river). Under these circumstances the observer may perceive the simultaneous presence of more than one velocity, within the same region of space, a phenomenon known as motion transparency. How this perceptual segregation is achieved in human vision is still not well understood and poses a challenge to conventional theories of motion perception that typically rely on a strict correspondence between one velocity vector and one spatial location (Hildreth, 1984; Yuille & Grzywacz, 1988).

Motion transparency has been extensively studied (Stoner & Albright, 1992; Braddick, 1993; Braddick, 1997), but in practice it is a difficult phenomenon to investigate in the laboratory because of its inherently subjective nature. Psychophysical studies have typically required participants to simply judge whether motion stimuli, composed of one or more direction(s) or speed(s), appear transparent (e.g., Smith, 1992; Qian, Andersen, & Adelson, 1994; McOwan & Johnston, 1996). Alternatively, participants may be asked to make judgments about the appearance (e.g., estimate the perceived direction) of only one of the moving components comprising a transparent stimulus (Marshak & Sekuler, 1979; Hiris & Blake, 1996; Kim & Wilson, 1996). However, as each observer is free to choose a response criterion to make these perceptual decisions, such techniques are inevitably susceptible to bias, thus limiting the conclusions that can be drawn. Nonetheless investigating the stimulus conditions that give rise to transparent motion perception, and the underlying neural mechanisms that mediate this phenomenon, is fundamental to our understanding of how motion can be used to delineate the layout of the visual environment.

The mechanisms that segregate moving objects appear relatively insensitive to the spatial scale of moving stimuli. For example, Smith (1992) investigated the maximum spatial frequency difference between the component gratings of a drifting plaid (e.g., Adelson & Movshon, 1982; Stoner & Albright, 1992) for which the overall pattern was still judged as coherent (rigid movement in a single direction) rather than transparent (two gratings sliding over each other). He found that at relatively high contrasts and slow drift speeds subjects could tolerate up to a four-octave difference in spatial frequency before the plaid was perceived as transparent. This insensitivity to spatial scale means that spatially broadband motion stimuli are well suited for probing the mechanisms underlying motion transparency.

Random-dot kinematograms (RDKs) have been widely used to investigate the inherent stimulus characteristics that elicit transparent motion (e.g., Gibson, Gibson, Smith, & Flock, 1959; Clarke, 1977; Andersen, 1989; Snowden, 1989). Smith, Curran, and Braddick (1999) investigated whether spatially overlapping RDKs with dot directions sampled from independent probability distributions give rise to transparent motion. They found that perceived transparency was related to the magnitude of the difference between the mean directions of the overlapping surfaces. However, adopting a similar approach, Hines-Turner and Braunstein (1994) did not find a straightforward relationship between changes in the distribution means and transparency perception. They instead showed that a better predictor of motion transparency is the ratio of distance between the two distributions (notch width) to the range of velocities present. It thus remains unclear whether absolute differences between the central tendencies of moving stimuli or the ratio of those differences to the range of velocities in the image are better predictors of motion transparency.

The range of velocities within the moving surface may themselves contribute to the perception of transparency. Masson, Mestre, and Stone (1999) measured speed-difference thresholds for motion transparent stimuli, where dots moved in the same direction but at different speeds. Participants were asked to report which RDK was composed of only two speeds compared to a “reference” RDK that consisted of five different speeds. They showed that thresholds for motion transparency were higher when dots moved at relatively fast speeds (above ∼8°/s). However, Masson et al. (1999) did not measure directly the relationship between observers' performance and transparent perception and used only a limited range of speeds. These findings are consistent with the results described by Meso and Zanker (2009), who suggested that gratings moving in the same direction are more likely to be perceived as transparent when comprised of large speed differences. However, early psychophysical work (Braddick, Wishart, & Curran, 2002) has shown that increasing dot speed from 1 to 10°/s did not cause changes in transparency perception. These discrepancies leave open the question of how the range of speeds in a moving surface changes transparency perception.

Motion-sensitive circuits in primary visual cortex (V1) and middle temporal cortex (MT) are likely neural substrates for transparent motion signals (e.g., Movshon, Adelson, Gizzi, & Newsome, 1985; Rodman & Albright, 1989; Snowden, Treue, Erickson, & Andersen, 1991; Stoner & Albright, 1992; Qian et al., 1994). Snowden et al. (1991) probed the circuits underlying motion transparency by recording extracellular responses in V1 and MT of awake behaving monkeys. They showed that responses to dot motion in the preferred direction of neurons were suppressed by the addition of overlapping dot motion in the antipreferred direction. This is consistent with recent work (Krekelberg & van Wezel, 2013), which showed bidirectional (transparent) motion suppresses responses of MT neurons at the preferred speed, but can also shift tuning to slower speeds.

In recent physiological work, McDonald, Clifford, Solomon, Chen, and Solomon (2014) measured the responses of subpopulations of neurons in MT to transparent dot motion. They found that “pattern cells” with unimodal direction selectivity to plaid motion and “component cells” with bimodal selectivity to plaid motion have distinct roles in transparent and coherent motion perception. Pattern cells respond to the constituent motion directions of moving dot fields, whereas component cells respond to the average direction of image motion. Constituent directions separated by at least 60° elicited bimodal responses in pattern cells, consistent with previous work (Qian et al., 1994; Treue, Hol, & Rauber, 2000). This strongly implicates the circuits that generate pattern selectivity in transparent motion perception (but see Xiao & Huang, 2015 for a different interpretation)—a long-standing prediction of Simoncelli and Heeger's (1998) model of motion processing in MT (but see Medathati, Rankin, Meso, Kornprobst, & Masson, 2017 for an alternative computational account).

Here we probe the underlying neural mechanisms by investigating how motion transparency arises from objects moving at different speeds within a visual scene. To prevent participants from adopting a subjective decision criterion when discriminating transparent motion, we used an “odd-one-out,” three-alternative forced-choice (3AFC) procedure. Transparent RDKs were created by sampling dot speeds, or directions, from two distributions separated by a notch that was systematically varied across different experimental conditions. Using this novel psychophysical procedure, we characterized the distributions of velocities in a scene that give rise to transparent motion perception. Our results suggest that transparency perception is driven primarily by relatively slow speeds, and does not arise when only fast speeds are present within a visual scene.

## Material and methods

### Participants

Four adults with normal or corrected-to-normal vision participated in the experiments. A minimum of three participants took part in each experiment described in the present study. FR was one of the authors, whereas the remaining participants (AG, DJH, and RJS) were naive to the purpose of this work. All were given extensive practice prior to formal data collection to familiarize themselves with the tasks.

### Apparatus and stimuli

Stimuli consisted of RDKs generated using custom software written in Python and the Psychopy package (Peirce, 2007). RDKs were presented on a cathode-ray-tube monitor (either LaCie Electron 22blue or IIyama Vision Master Pro 514) with a spatial resolution of 1,280 × 1,024 pixels, at a refresh rate of 75 Hz. The luminance of the monitor was gamma-corrected with a spot photometer (LS-110; Konica Minolta, Mississauga, Canada) and internal look up tables. The viewing distance was 76.3 cm, such that one screen pixel subtended 1.35 arc min of visual angle.

Each of the images comprising a motion sequence consisted of 226 dots (dot luminance 0.05 cd/m^2^) randomly displayed within a circular window (diameter 12°) on a uniform luminance background (25 cd/m^2^). The diameter of each dot was 0.1° and the dot density was 2 dots/deg^2^. Dots that fell outside the circular window were redrawn on the opposite side of the window. Continuous apparent motion was generated by presenting the images consecutively at an update rate of 18.75 Hz, which is comparable to previous work using RDKs (e.g., Williams & Sekuler, 1984; Watamaniuk, Sekuler, & Williams, 1989; Watamaniuk & Sekuler, 1992; Webb, Ledgeway, & McGraw, 2007; Webb, Ledgeway, & McGraw, 2010; Webb, Ledgeway, & Rocci, 2011). Each RDK was generated anew on each trial and consisted of 10 images presented for a total duration of 530 ms.

Where speed was manipulated, individual dot speeds (and hence spatial displacements) were sampled with replacement from an underlying speed distribution on every positional update. Thus, the speed of a dot was not constant throughout the duration of each RDK, but underwent a random walk, so individual dots were not assigned a single unique speed. This is important as it ensures that tracking the extended trajectories of individual dots is not necessary to extract the highest speeds present in the underlying standard and comparison distributions.

### Procedure

To ensure that participants could reliably perceive the direction of apparent motion for all the dot displacement magnitudes (speeds) used in this study, we used a single-interval, forced-choice task and measured the maximum displacement limit (D_max_) for RDKs. D_max_ is a measure of the maximum distance over which dots can be displaced on consecutive images and still give rise to reliable judgments of motion direction (Braddick, 1974). The RDKs were identical to those used in the main experiments, with the exception that all dots in the display were displaced by the same amount on each positional update and in the same direction (either leftwards or rightwards, chosen at random on each trial). Participants identified whether the RDK moved leftwards or rightwards on each trial and performance was measured for each of a range of dot displacements from 27 to 162 arc min.

**Figure 1:**
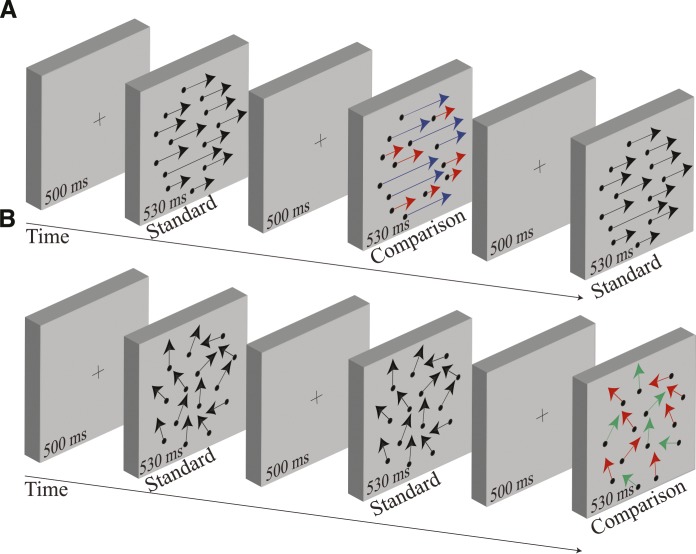
Schematic representation of the 3AFC task used to measure transparent motion perception. The comparison RDK was randomly presented only in one of the three intervals. Participants were required to discriminate which of the three RDKs was different (in speed or direction). (A) Schematic illustration of the RDKs used to investigate speed transparency perception. The length of the arrows indicates the speed of the dots. In the standard RDK, dots were sampled from a uniform speed distribution. The distribution of speeds was identical for both standard stimuli presented on each trial but the RDK samples used in the two standard intervals were different. The comparison RDK was composed of dots drawn from two distributions, set apart by an interval in speed (notch width). The direction of the dots was identical for all three RDKs presented on each trial. (B) Schematic representation of RDKs used to test transparency perception based upon direction differences. The standard RDK was composed of dots sampled from one uniform distribution of directions (different samples used for each standard interval), whereas the comparison RDK consisted of dots drawn from two distinct direction distributions, separated from each other by an interval in direction (directional gap width). The speed of the dots was identical for all three RDKs presented on each trial. Different colors are used for illustrative purposes only and indicate dot directions (or dot speeds) sampled from different distributions.

In a three-alternative, odd-one-out forced-choice task, participants were instructed to indicate which interval contained the RDK that differed in speed or direction from the other two (see Figure 1). The three intervals consisted of RDKs presented in a random temporal order, separated by interstimulus intervals of 500 ms. Two intervals, containing the standard RDK, consisted of a single dot field with speeds or directions sampled from the same distribution. The distributions were identical for both standard stimuli presented on each trial but the RDK samples used in the two standard intervals were different. The other interval, contained the comparison RDK, consisted of two spatially overlapping dot fields sampled from two distinct distributions. The total range of dot speeds (or directions) was the same for both standard RDKs and the comparison RDK.

Participants correctness at identifying the odd-one-out depended upon their ability to segregate two distinct objects (or components) moving, at the same time, across the same spatial region of the visual field in the comparison. This simple experimental design provides a method for investigating perceived motion transparency without participants making any subjective judgment about the motion distributions. Observers' ability to segregate superimposed motion patterns was expressed as the proportion of correct responses as a function of the physical separation (speed notch width or directional gap width) between the two motion distributions that made up the comparison. Participants did not receive feedback on the correctness of their responses. They completed a minimum of 280 trials for each experimental condition.

## Results

### Maximum spatial displacement limit

The maximum spatial displacement limit (D_max_) supporting reliable motion perception (i.e., giving rise to at least 75% correct responding) was 100 arc min (data not shown). This displacement, if maintained, at an update rate of 18.75 Hz corresponds to a dot speed of 31.25°/s and none of the dots in the following experiments exceeded this value.

### Experiment 1: Motion transparency when moving images have distinct speed components

**Figure 2:**
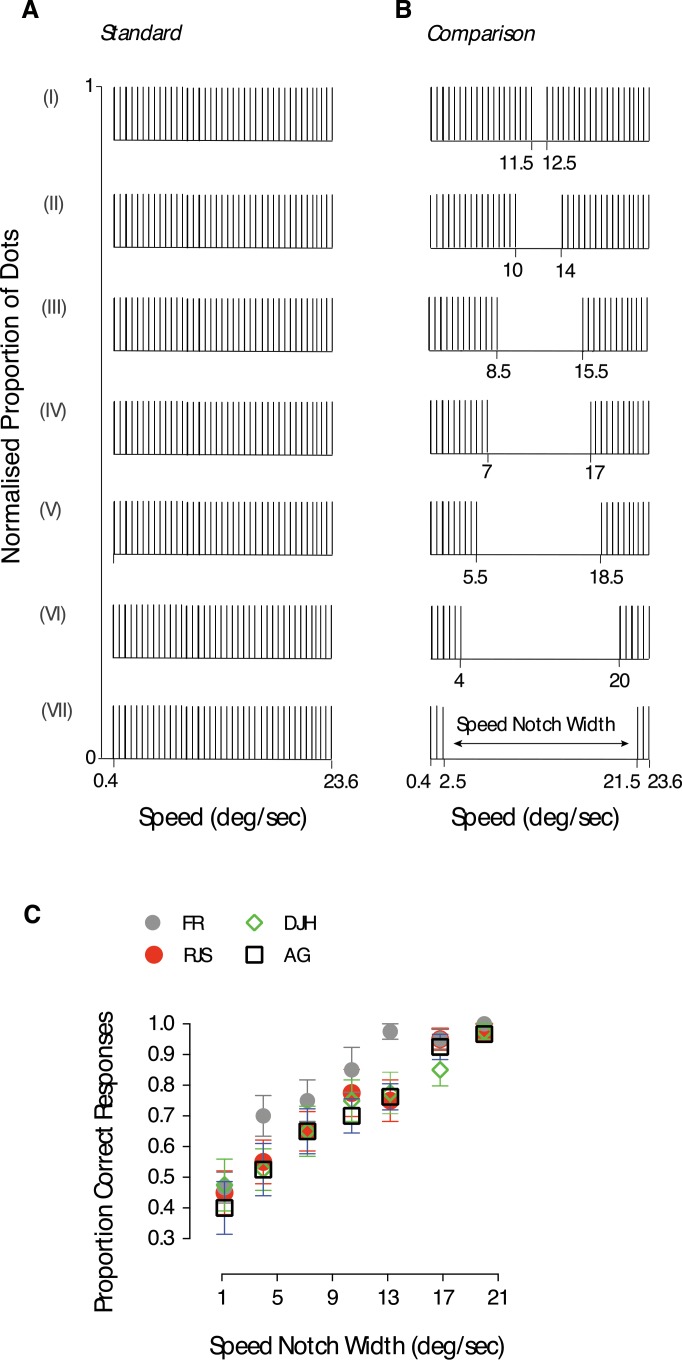
Discrimination of transparent motion is driven by visual speed components. (A) The standard RDK was identical throughout the conditions (I-VII). (B) The comparison RDK consisted of two independent speed distributions separated from each other by an interval in speed (speed notch width). Seven speed notches were used to test transparency perception (either 1°/s, 4°/s, 7°/s, 10°/s, 13°/s, 16°/s, or 19°/s). (C) The proportion of correct responses is plotted as a function of the notch width between the speed distributions used to construct the comparison RDK. Symbols indicate individual data points of four subjects. Observers' performance improved as the notch width between the two speed distributions increased. Error bars represent ±1 SEM.

We first investigated how the speeds of superimposed moving dot fields contribute to transparent perception. The standard RDK (Figure 1A) was always composed of one field of dots sampled (with replacement) from a uniform speed distribution spanning 23.2°/s (Figure 2A). The comparison RDK (Figure 1A) consisted of two spatially intermingled dot fields, sampled from two speed distributions (Figure 2B). The total range of speeds employed for both the standard and the comparison RDKs (0.4°/s to 23.6°/s) was the same. On each trial, both the standard and the comparison RDKs had the same direction, randomly selected from a range spanning 360°, and differed only in speed. We randomly varied the separation in speed (speed notch width) between the two distributions to be either 1°/s, 4°/s, 7°/s, 10°/s, 13°/s, 16°/s, or 19°/s (Figure 2B). In all conditions, dot speeds were sampled at 1°/s intervals and the comparison stimulus and the standard RDK had the same global arithmetic mean speed (12°/s).

Figure 2C shows four participants' ability to discriminate transparent from coherently moving surfaces. The detection of the odd-one-out is expressed as the proportion of correct responses as a function of the speed notch width (separation between the two distributions from which the comparison RDK dot speeds were sampled). Participants' ability to detect the spatially superimposed distributions improved with increasing speed notch width. With a notch width of 19°/s, participants identified the comparison RDK correctly on over 90% of trials, demonstrating that a distinct demarcation between the speeds present within a stimulus contributes to the likelihood of it being perceived as transparent.

### Experiment 2: Summary statistics and motion transparency perception

**Figure 3:**
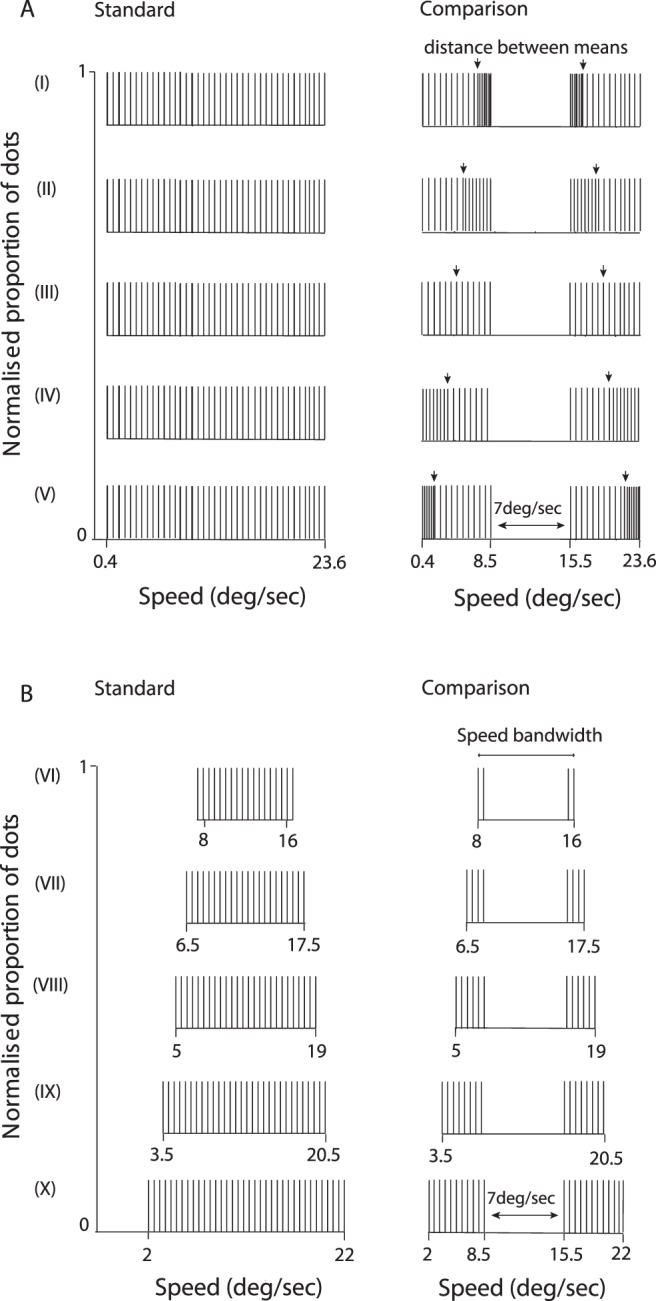
Speed distributions employed to test how motion statistics affect transparency perception. The speed notch characterizing the comparison RDK was constant (7°/s). (A) The standard RDK was composed of dots sampled from a uniform distribution. The distance between the (local) means of the distributions employed to draw the comparison RDK was systematically manipulated. (B) Distributions designed to explore the relationship between the range of speeds used (either 8°/s, 11°/s, 14°/s, 17°/s, or 20°/s) and perceived motion transparency.

**Figure 4:**
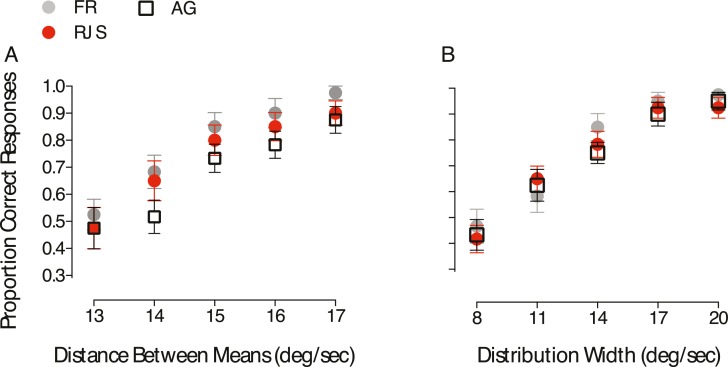
Motion statistics affect the perception of overlapping (transparent) surfaces. (A) Proportion of correct responses as a function of the differences between the (local) means of the comparison RDK. (B) Observers' performance as a function of the range of speeds presented. The gap width that set apart the comparison RDK distributions was always constant and equal to 7°/s. Error bars represent ±1 SEM.

Our aim here was to explore the relationship between stimulus speed statistics (e.g., mean and variance) and transparency perception. To establish the role played by the central tendency of the speed of a moving surface in transparency perception, we manipulated the distance between the mean speeds (either 13°/s, 14°/s, 15°/s, 16°/s, or 17°/s) of the two comparison distributions by changing their sampling density (see Figure 3A). Dot speeds were sampled at either 1°/s, 0.2°/s, or 0.6°/s intervals. The range of each of the two comparison distributions was fixed at 8.1°/s and the notch width separating them was fixed at 7°/s. The global arithmetic mean of the comparison RDK, computed over all dots in the image, was 12°/s with an overall range the same as the standard. The direction of motion was chosen as described in Experiment 1. Figure 4A shows the relationship between the participants' ability to perceive transparent motion and the distance between the (local) means of the two speed distributions in the comparison RDK. As the distance between the means increases, participants' performance clearly improves. Consistent with previous work (Smith et al., 1999), motion transparency perception was driven by differences between the means that characterized the overlapping motion patterns.

To assess whether the overall speed range present in the image modulates transparency perception, we varied the speed bandwidth (either 8°/s, 11°/s, 14°/s, 17°/s, or 20°/s) of the standard and comparison RDKs (Figure 3B). The standard stimulus consisted of dots sampled from one uniform distribution, whereas the comparison RDK was composed of dots drawn from two speed distributions with a fixed notch width of 7°/s. Figure 4B shows that when the speed bandwidth was increased (but the speed notch width was held constant) participants' ability to correctly identify the comparison RDK also improved. Contrary to the results reported by Hines-Turner and Braunstein (1994), this finding suggests that participants are better at detecting motion transparency when the ratio of the notch width to the full range of speeds decreases.

### Experiment 3: Transparent motion perception at “slow” and “fast” speeds

**Figure 5:**
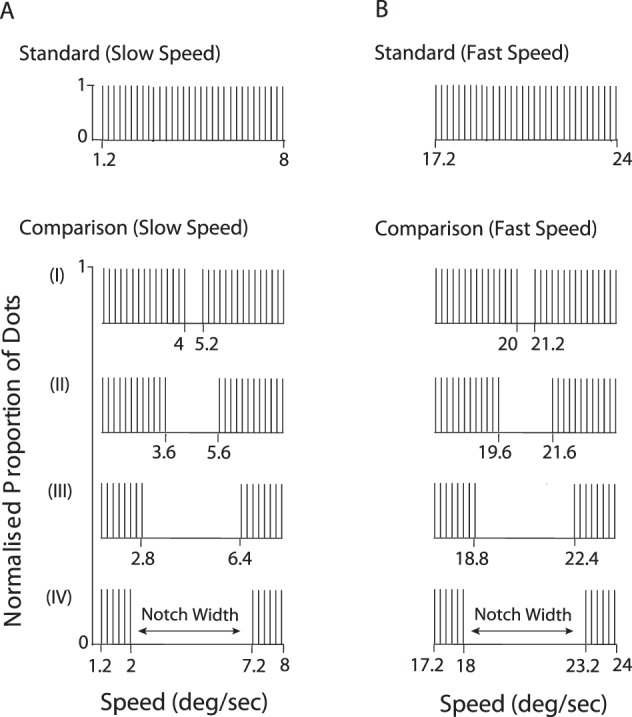
Illustration of the speed distributions designed to test whether or not transparency perception depends on the absolute speeds used (either slow or fast speeds). (A) Speed distributions generated to test the relationship between motion transparency perception and relatively slow speeds. (B) Dot distributions employed to investigate transparency perception with relatively fast dot speeds. For each notch width (I-IV), the distance between the (local) means of the comparison RDK distributions was identical across the two ranges of speeds used.

Snowden (1990) previously proposed that distinct mechanisms might account for motion perception at relatively “slow” and “fast” speeds. How this hypothesis holds up with transparent motion stimuli, using an objective forced-choice task, has never been tested. Here we compared transparency motion perception using speed distributions centered on relatively slow (Figure 5A) and fast (Figure 5B) speeds. The slow speed standard ranged from 1.2°/s to 8°/s and the fast speed standard ranged from 17.2°/s to 24°/s (Figure 5B). The overall range of speeds was the same for both standard and comparison RDKs. The notch width of the comparison varied (1.2°/s, 2°/s, 3.6°/s, or 5.2°/s) but the distance between the means of the two distributions was kept constant. The comparison and the standard RDKs were characterized by an identical global arithmetic mean speed (4.6°/s for the slow speed conditions and 20.6°/s for the fast speed conditions).

**Figure 6:**
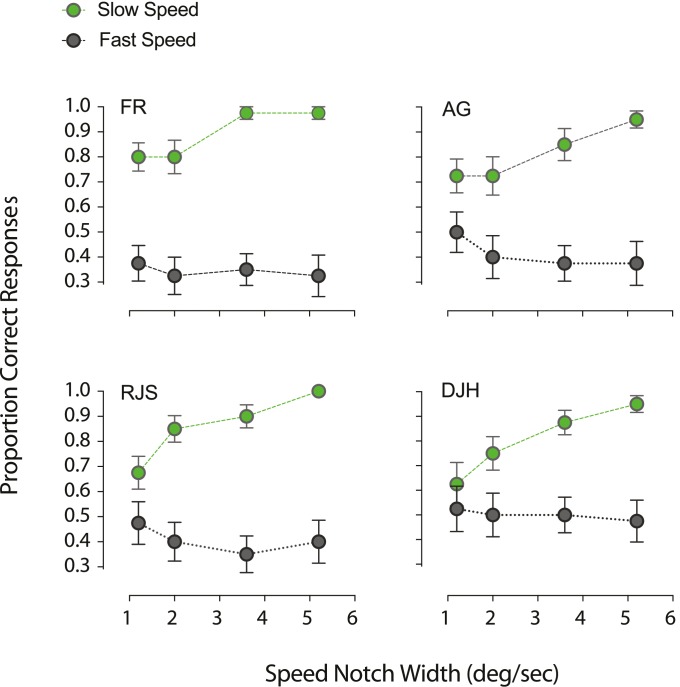
Observers' ability to discriminate superimposed RDKs depends of the range of speeds used. The probability of discriminating transparent motion patterns dropped close to chance levels when relatively fast dot speeds were employed. Transparency perception was driven by slow speeds. Error bars represent ±1 SEM.

Figure 6 shows participants' discrimination of transparent motion when dot speeds were sampled from distributions centered on slow (green symbols) and fast speeds (black symbols). Participants almost always correctly discriminated the comparison from the standard RDKs when the moving image was composed of relatively slow speeds. In contrast, at relatively fast speeds, participants were unable to discriminate the RDKs and were close to chance (33.3% correct). This strongly indicates that motion transparency is primarily observed at slow speeds, supporting the notion that distinct mechanisms may mediate the perception of different image speeds.

### Experiment 4: Velocity-dependent transparent motion perception

**Figure 7:**
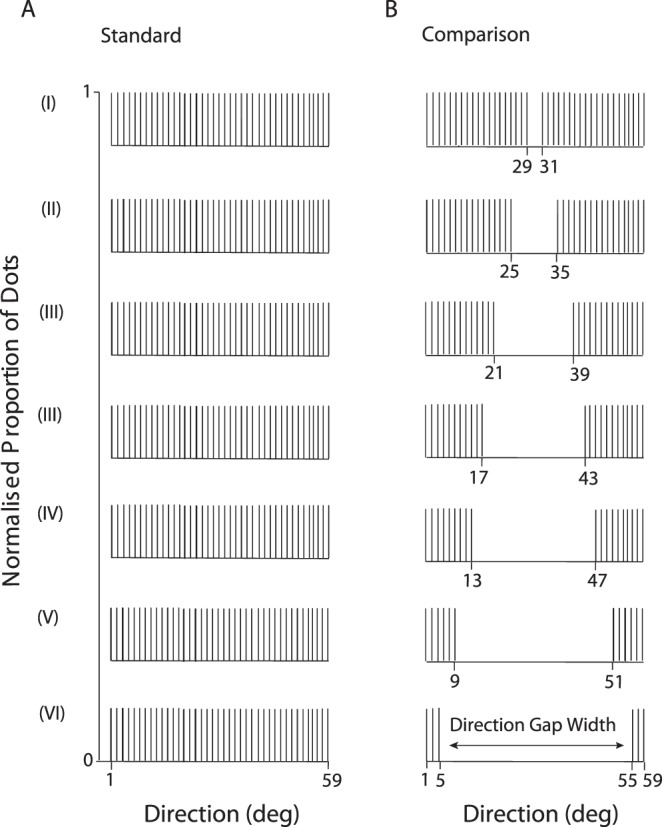
Schematic representation of the direction distributions designed to explore transparency perception elicited by differences in directional components. The standard RDK was the same throughout the conditions (I-VI). The dots of the comparison RDK were sampled from two direction distributions and set apart by an interval in direction (directional gap width) which was either 2°, 10°, 18°, 26°, 34°, 42°, or 50°.

We tested how different speeds affect transparency perception of RDKs composed of distinct, but superimposed, directional components. The standard RDK (Figure 1B) always consisted of dots drawn, with replacement, from a single uniform direction distribution spanning 58° (Figure 7A). The dots of the comparison RDK (Figure 1B) were drawn from two independent distributions, set apart by a gap in direction (directional gap width) that was either 2°, 10°, 18°, 26°, 34°, 42°, or 50° (Figure 7B). All the dot directions were sampled at 1° intervals. The overall mean (30°) and the range of directions used were the same for both the comparison and the standard RDKs. Speed was fixed in three different conditions (4°/s, 13°/s, or 22°/s).

**Figure 8:**
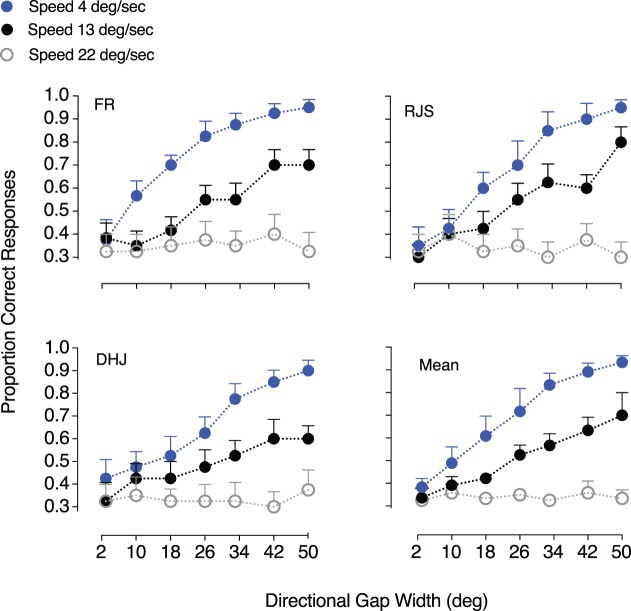
Relationship between transparent motion perception and superimposed velocity fields. Data are expressed in terms of proportion of correct responses as a function of varying the directional gap width between comparison RDK distributions. Observers' performance was measured separately for three different speeds (either 4°/s, 13°/s, or 22°/s). The ability to distinguish between superimposed translating motion surfaces was affected by both the directional gap width and the absolute speed of the dots. The average performance across all observers, computed by combining the three observers' data, is shown in the bottom right panel. Error bars represent ±1 SEM.

Our results show that transparent motion perception of distinct direction distributions was different at different speeds (Figure 8). For the largest directional gap width of 50°, participants correctly discriminated the comparison (transparent) RDK from the standard RDKs with a high probability (>90%) when dots were moving slowly (4°/s). However, performance decreased markedly as speed increased, and was close to chance when dots moved at 22°/s. This result is consistent across participants and demonstrates that absolute image speed modulates the strength of perceived transparency arising from directional differences between objects or surfaces within a scene.

## Discussion

Our aim was to characterize the speed mechanisms underlying motion transparency perception in human vision. We used an objective, forced-choice, odd-one-out measurement of random-dot motion transparency perception—a task that did not depend upon participants adopting an arbitrary, subjective response criterion. Our results reveal that transparent motion perception is strongest at relatively slow speeds, and rarely emerges at fast speeds. Furthermore stimulus-based characteristics, such as the separation between the means of the superimposed distributions and the range of speeds presented within an image, modulated transparent perception of moving surfaces.

The role of speed information in mediating transparency perception has received relatively little attention. Previous studies investigating transparency perception have focused predominantly on how directional differences, between spatially overlapping patterns, can give rise to the percept of multiple moving objects at the same location in the visual field (e.g., Stoner & Albright, 1992; Braddick, 1997). Our results suggest that speed has a pivotal role to play in motion transparency. Participants are able to use the speed profile of moving objects to integrate local motion signals into different global percepts, and simultaneously segregate local information into distinct objects. Increasing the separation (notch width) between two spatially intermingled speed distributions improves participants' discrimination of two independently moving surfaces.

We sought to identify the critical features of speed distributions that determine the likelihood of perceiving motion transparency within a visual scene. Smith et al. (1999) suggested that transparency perception is not guided by the size of the critical gap (or notch width) between distributions, but rather by the difference between their means. However, the directional gap width between the pair of distributions employed by Smith et al. (1999) covaried with the relative distance between means. We explored if the difference between the means of the two distributions (comprising the transparent RDK) affects performance on an objective transparency-based task even when the speed notch width is constant. Our results are consistent with those of Smith et al. (1999) in that participants' discrimination of two overlapping motion surfaces improved with increasing distance between their mean speeds.

Our results, however, do not agree with those of Hines-Turner and Braunstein (1994). They showed that differences between the mean speeds of two moving surfaces did not affect transparency perception. These authors used RDKs that moved in a unitary direction and were composed of dot speeds sampled from distinct distributions, separated by a speed notch. They found that the range of speeds (bandwidth of the speed distributions) is critical for perceiving overlapping moving surfaces. When the ratio of the distance between the means to the full range of velocities dropped below 30%, they showed that participants were unable to identify the presence of the two superimposed motion patterns. Although we also found discrimination performance varied as a function of the distribution width, discrimination performance actually declined as the ratio of notch width to the range of speeds increased (dropping close to chance levels when this ratio exceeded 0.6). This is the opposite of that described by Hines-Turner and Braunstein (1994). These discrepant results might be due to methodological differences between the two studies. For example, Hines-Turner and Braunstein (1994) used a subjective task to study transparency perception. That is, subjects were presented with pairs of spatially superimposed velocity distributions and reported the impressions of apparent depth arising from the moving dots. That is, whether the dots appeared to fill a single volume, or two volumes separated along the line of sight in depth. In contrast, the current study utilized an objective, forced-choice odd-one-out task ensuring that individual differences in response criteria and discrimination performance were not conflated.

One potential concern with our design is that the number of dots assigned to each speed covaries with distribution width and that this could, in part, explain some of our results (e.g., those shown in Figure 4). However, we think it unlikely that this has a marked influence on our results for the following reasons. In Experiment 1, performance improved as the speed notch width increased, and hence also the number of dots/speed, was increased (see Figure 2B and C). However, in Experiment 2 the opposite effect was found: Performance deteriorated as the distribution width decreased, even though the number of dots/speed increased accordingly (see Figures 3B and 4B). This provides good evidence that under the conditions of our study the number of dots attributed to each speed cannot, by itself, play a major role in the pattern of results found.

Although we showed that discrimination of transparent motion varied as a function of motion statistics, these cues might not be the only factor driving motion transparency perception. For example, Masson et al. (1999) found that speed-difference thresholds for transparent stimuli varied with the range of speeds presented. In a companion paper, Masson's group also found that motion transparency perception depends upon the spatial distribution of speeds within a moving pattern (Mestre, Masson, & Stone, 2001). However, Masson et al. (1999) did not control the distance between the means, nor the speed bandwidths of the superimposed motion patterns. Moreover, transparency perception was not measured with an objective task, but required participants to identify which of two intervals contained two speeds. This assumes (but is not verifiable) that two transparent surfaces could always be perceived when only two speeds were presented within the same spatial region.

Our objective odd-one-out task assumes that the participants base their judgments on the perception of transparent motion, although we cannot confirm this as transparency is itself a purely subjective phenomenon. This is not an unreasonable assumption, however, since we have taken great care to match the standard and comparison RDKs on both the range of speeds present in the images and also the global mean dot speed. Even when the notch width is relatively large (e.g., 19°/s) subjects could not base their decision on (say) a strategy of choosing the interval that contains dots that move slowly, since the presence of slowly moving dots is common to all three stimuli (the two standards and the comparison RDK) presented on each trial. Subjects instead would need to identify some other characteristic that differs between the stimuli, such as the presence of the notch in the comparison RDK which we assume leads to transparency.

It has been proposed that at least two independent speed-tuned mechanisms underlie global motion processing. Snowden (1990) investigated the masking effect that a vertically moving dot field has on displacement detection of horizontally moving dot patterns. On the basis of his findings, he posited two speed-tuned global motion mechanisms: one selective for relatively slow speeds, and one for faster speeds. However, Verstraten, Fredericksen, van Wezel, Boulton, and van de Grind (1996) subsequently explored the perceived orthogonal-masking effect and found less evidence for speed selectivity than that reported by Snowden (1990), questioning the need for independent mechanisms. Another study that bears directly on this issue was conducted by Edwards, Badcock, and Smith (1998). They used RDKs in which a proportion of the dots moved coherently in one direction (signal) and the remaining dots (noise) moved randomly. Edwards and colleagues showed that the ability to identify the global direction of signal dots moving at a relatively slow speed (1.2°/s) was affected only by additional noise dots moving at relatively slow speeds (<4.8°/s). In contrast, when signal dots moved at a relatively fast speed (10.8°/s), observers' performance was most impaired by the presence of additional noise dots moving at a similar speed. This finding suggests that at least two independent speed-tuned mechanisms might underlie global motion processing.

To probe these putative speed-tuned mechanisms, we measured motion transparency perception over two different speed ranges: slow speeds (1.2°/s to 8°/s) and fast speeds (17.2°/s to 24°/s). Our results revealed that motion transparency is only perceived in the slow speed range, consistent with the notion of independent speed-tuned mechanisms (cf. Snowden, 1990; Edwards et al., 1998). When we tested how different speeds affect transparency perception composed of distinct directional components, motion transparency was also only perceived at slow speeds (see Figure 8). This suggests that absolute image speed modulates the strength of perceived transparency arising from directional differences between objects or surfaces within a scene.

Studies of the motion aftereffect also fit with the existence of at least two speed-tuned channels (Anstis, Verstraten, & Mather, 1998; Mather, Verstraten, & Anstis, 1998). Transparent adapting stimuli typically lead to a unidirectional aftereffect (Mather & Moulden, 1980), but when one motion pattern is characterized by a slow speed and the other is moving at a much faster speed, adaptation to transparency can lead to a transparent motion aftereffect (van der Smagt, Verstraten, & van de Grind, 1999). Similar work on binocular motion rivalry has found an absence of rivalry between relatively slow and fast motions (van de Grind, van Hof, van der Smagt, & Verstraten, 2001). Our results support the notion of independent speed-tuned mechanisms and suggest that perceptual transparency might be governed by a mechanism predominantly tuned to slow speeds, consistent with the tuning of some MT neurons to bimodal motion (Krekelberg & van Wezel, 2013).

The circuits that generate motion pattern-selectivity within MT are a putative neural mechanism for transparent motion perception (McDonald et al., 2014, but see Xiao & Huang, 2015). Our results indicate that the mechanism(s) driving transparency perception should be tuned to absolute speed, with a preference for slower speeds. These characteristics of motion transparency perception are more challenging to reconcile with the selectivity of pattern neurons in MT, which tend to prefer faster random-dot motion (Wang & Movshon, 2016). More work is thus required to clarify the relationship between pattern selective circuits in MT and transparent motion perception.

In summary, our results show that transparency perception is driven primarily by relatively slow speeds (below 8°/s), and rarely present at faster speeds (above 17.2°/s). Transparent perception of moving surfaces is modulated by stimulus-based characteristics, such as the separation between the means of the overlapping velocity distributions or the range of speeds presented within an image. Finally, our work illustrates the utility of using objective, forced-choice methods to study the mechanisms underlying motion transparency perception.
